# Digital interventions for supporting alcohol abstinence in aftercare – a systematic review

**DOI:** 10.1016/j.invent.2025.100832

**Published:** 2025-05-15

**Authors:** Luana Lenzi, Aaliya Ibrahim, David Brough, Alexander Thompson

**Affiliations:** a Manchester centre for Health Economics, The university of Manchester. Oxford Road, Manchester M13 9PL, UK; b Manchester Medical School, University of Manchester, Manchester, UK; c Mind Healthcare, Elizabeth House, The Pier, Southgate, Wigan WN3 4EX, UK

**Keywords:** Alcoholism, Alcohol use disorder, Alcohol abstinence, Behaviour change, Digital intervention, mHealth, eHealth

## Abstract

**Background and aims:**

Alcohol Use Disorders (AUD) are associated with numerous negative health and societal consequences. Relapse is common among individuals with AUD following discharge from treatment programs, often due to a lack of continuing care and barriers to accessing in-person interventions. Digital interventions may have the potential to overcome these barriers. This systematic review aims to assess the efficacy of digital interventions in supporting abstinence following AUD treatment.

**Methods:**

We searched the databases *Embase, Medline,* and *APA PsycInfo* for randomized controlled trials (RCTs) that evaluated digital interventions designed to support alcohol-dependent individuals to maintain abstinence after discharge from treatment programs. Studies in which participants were not abstinent at the time of randomization were excluded.

**Results:**

Eleven studies were identified, with interventions including text messages, smartphones apps, wireless breathalysers, telephone-based support, and e-books. Four studies (2 using apps and 2 using supportive text messages) reported statistically significant results in prolonging abstinence. However, one intervention using a cue exposure therapy (CET) app found increased relapse rates in all groups. The risk of bias across studies ranged from moderate to high.

**Conclusion:**

There is insufficient evidence to support the efficacy of digital interventions in maintaining abstinence after AUD treatment discharge. While digital interventions may improve the accessibility and uptake of aftercare services to prevent relapse, further research is needed.

## Highlights

• The evidence regarding digital intervention to prolong abstinence are limited.
• Smartphone apps and text messages interventions show promising results.
• Age, sex and AUD treatment may influence the usability and effectiveness of digital interventions.

## 1 Introduction

Alcohol Use Disorders (AUD) are a prevalent and harmful neuropsychiatric condition that accounts for over 5 % of the global disease burden, resulting in millions of deaths annually and placing a significant economic burden on individuals and healthcare systems (Gushken et al., 2025; Bickel et al., 2024). AUD has been linked to the development of >60 medical conditions, including life-threatening illnesses such as liver cirrhosis and cancer (O'Donnell et al., 2022; Whiteford et al., 2013), and is also associated with broader societal consequences such as motor vehicle accidents, criminal activity and domestic violence (Carvalho et al., 2019). The underlying causes of AUD are not fully understood; however, it is believed that factors such as the accessibility and promotion of alcohol in an individual's environment, as well as cognitive and genetic predispositions, may contribute to its development (Verhulst et al., 2015). Additionally, AUD frequently co-occurs with mental health disorders, with an estimated two out of three people with alcohol use problems also experiencing conditions such as depression or anxiety (Deady et al., 2014; Schuckit and Hesselbrock, 2004).

There are two primary treatment modalities for managing AUD: inpatient care, which involves staying at a residential facility or hospital to receive 24/7 medical and psychological support, and outpatient care, which allows individuals to live at home while attending scheduled therapy or medical appointments at a clinic or treatment centre. Each option has distinct characteristics, benefits, and suitability depending on the severity of the condition and the individual's specific needs (Mar et al., 2023). In terms of treatment interventions for AUD, these can be grouped in pharmacological treatments, such as naltrexone, disulfiram and acamprosate, support programs, such as Alcoholics Anonymous (AA), and behavioural therapies such as Cognitive-behavioural therapy (CBT), motivational interviewing (MI), and contingency management (Gushken et al., 2025; Carroll and Kiluk, 2017). Typically, patients are encouraged to try non-pharmacological therapies first, as they provide individuals with a supportive environment and network to help maintain abstinence. Despite these interventions, many individuals with AUD struggle to achieve and sustain abstinence. Although long-term data on sobriety and relapse rates are somewhat limited and variable, studies suggest that approximately 60 % of individuals treated for AUD relapse within the first six months of treatment (Nguyen et al., 2020). Furthermore, around 30 % of individuals with advanced liver disease related to AUD relapse while awaiting a liver transplant (Suffoletto and Scaglione, 2018; DeMartini et al., 2018; DiMartini et al., 2010). This difficulty may stem from a variety of factors. Globally, only 21.9 % of people with AUD receive treatment, with barriers such as geographical distance limiting access to in-person interventions, social stigma deterring people from seeking help, and a shortage of specialists to deliver treatments like CBT (O'Donnell et al., 2022; Carvalho et al., 2019).

While abstinence remains the primary goal of treatment, there is often insufficient continuing care to support individuals after completing a rehabilitation program, contributing to high relapse rates (McLellan et al., 2003; Bandawar et al., 2018). Advances in digital technology present an opportunity to expand access to care and enhance public health outcomes by delivering continuing-care interventions on a broader and more accessible scale than traditional face-to-face methods (O'Donnell et al., 2022; Suffoletto and Scaglione, 2018; Bandawar et al., 2018). Digitally delivered interventions have the potential to address barriers such as geographic distance, resource scarcity, and fear of stigma (O'Donnell et al., 2022; McLellan et al., 2003; Bonfiglio et al., 2022). For example, mobile phone-based interventions can provide individuals with AUD access to support during critical moments, which is often impractical with in-person services (O'Donnell et al., 2022; Suffoletto and Scaglione, 2018). The COVID-19 pandemic further underscored the need for digital technologies to bridge the gap between services and their users (Bonfiglio et al., 2022).

A large-scale systematic review of 57 studies conducted in 2017 found evidence suggesting that digital interventions were associated with reduced alcohol consumption (Kaner et al., 2017). Compared to participants receiving no or minimal intervention, those in the digital intervention groups experienced, on average, one fewer drinking day per month, fewer binge drinking episodes, and consumed fewer units of alcohol per drinking session. However, in a subset of five smaller studies comparing digital interventions directly to face-to-face interventions, no significant difference in alcohol consumption was observed between the groups (Kaner et al., 2017). Despite mixed results, promoting multiple modalities of treatment delivery is crucial to increasing service accessibility and enhancing health outcomes (Bonfiglio et al., 2022).

Although there are some evidence supporting digital interventions for substance use disorders more broadly, research specifically evaluating their efficacy in reducing alcohol consumption and improving mental health outcomes for alcohol-dependent individuals after discharge remains limited. This systematic review aims to gather evidence on the effectiveness of digital interventions in supporting individuals in maintaining sobriety after AUD treatment discharge.

## 2 Methods

This systematic review was performed in accordance with the Preferred Reporting Items for Systematic Review and Meta-Analyses (PRISMA) guidelines (Page et al., 2021; Cohen et al., 2021). A protocol was pre-registered on PROSPERO (CRD42023418278).

### 2.1 Study selection

A systematic search was performed using the electronic databases Embase, Medline and APA PsycInfo using the Ovid search interface from inception date to 28th January 2025. There was no language restriction.

Key terms such as ‘addiction’, ‘abstinence’, ‘digital technology’, their synonyms and associated terms were combined using Boolean operators AND and OR. Manual searches were conducted using cross citation methods but did not retrieve any additional studies. All identified references were collated into Endnote™ version 20, where duplicates were removed. Titles and abstracts of the deduplicated studies were independently screened by two reviewers using the software Rayyan (Ouzzani et al., 2016). Discrepancies were initially discussed between the reviewers, and if consensus could not be reached, a third reviewer would be consulted; however, this step was not required during the screening process. The full texts of the included studies were then retrieved and screened for more accurate assessment of inclusion.

### 2.2 Eligibility criteria

Randomized controlled trials (RCTs) of digital interventions used to support maintaining sobriety after AUD treatment discharge compared with in-person care, treatment as usual or no intervention were included in this systematic review. Digital interventions could comprehend any form of technology such as text messages, telephone calls or mobile applications. Participants had to be abstinent at the time of randomization. Study protocols, dissertations, conference abstracts and qualitative research were excluded.

### 2.3 Outcome

The outcome of interest was data reflecting the efficacy of digital interventions in sustaining sobriety. This could be measured through indicators such as the number of days of alcohol abstinence or time to first drink. Both absolute measures (e.g., counts) and relative measures (e.g., percentages, averages, or scores) were extracted.

### 2.4 Data extraction and synthesis

A standardized Excel form was used for data extraction. Two independent reviewers extracted studies general data such as author, year and country of publication; baseline characteristics of the studies including participants number, age and sex; and data concerning the intervention included the treatment received at baseline such as the intervention and control conditions, the primary outcomes of the study and results related to the primary outcomes of each study. A meta-analysis was not feasible due to the heterogeneity of the studies. Instead, study characteristics and results were analysed and reported using descriptive statistics where applicable and a narrative synthesis has been presented.

### 2.5 Quality assessment

The risk of bias was measured using the Cochrane Collaboration's tool for assessing the risk of bias in randomized trials of interventions - RoB 2.0 (*Cochrane Handbook for Systematic Reviews of Interventions Version 6.0 (updated* 2020), 2020). The following sources of bias were evaluated: selection, performance, detection, attrition, and reporting bias. Evidence was classified as low risk of bias, some concerns or high risk of bias.

## 3 Results

### 3.1 Study selection

**Fig. 1:**
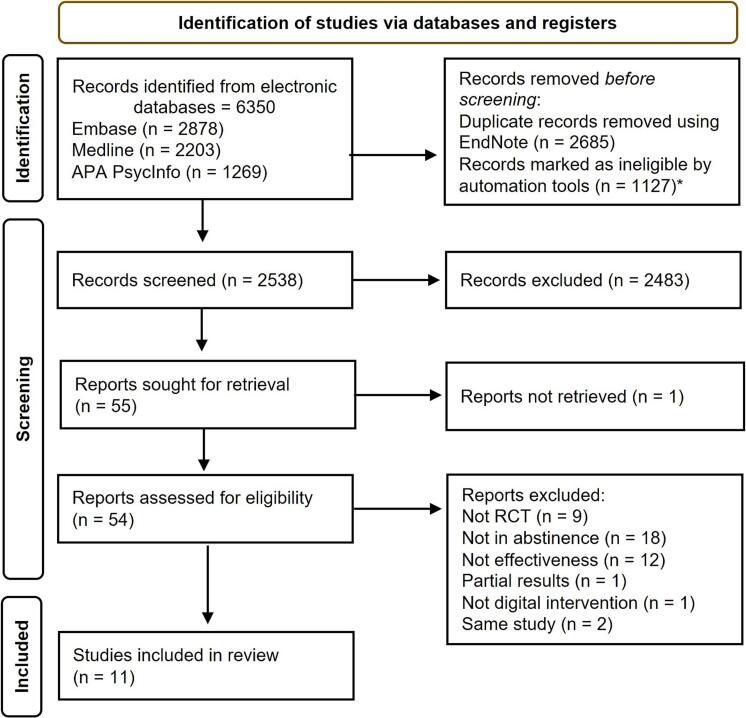
– PRISMA flow chart of the study selection. * Protocols (*n* = 265); Conference abstracts (*n* = 862).

This systematic review identified 6350 potential studies. After deduplication and the automatic exclusion of ineligible studies, 2538 references were screened. Following a thorough review of titles and abstracts, 54 studies were selected for full-text assessment, of which 11 met the inclusion criteria. The study selection process is summarized in Fig. 1. Two studies (Agyapong et al., 2013; Glass et al., 2017) were derived from the same trial; therefore, only the primary paper describing the effects of the digital intervention on abstinence after AUD treatment discharge was included. Studies were excluded if they involved interventions for non-abstinent patients at randomization, feasibility and acceptability studies without efficacy outcomes, or non-RCTs.

### 3.2 Study characteristics

All included studies were randomized controlled trials (RCTs), with their main characteristics summarized in Table 1. Three studies were conducted in Ireland (Agyapong et al., 2012; Farren et al., 2022; O'Reilly et al., 2019), two in the USA (Buono et al., 2023; Gustafson et al., 2014), two in Switzerland (Graser et al., 2021; Haug et al., 2015), and the remaining studies took place across multiple countries (Agyapong et al., 2018; Esselink et al., 2024; Lucht et al., 2021; Mellentin et al., 2019). The 11 RCTs included a total of 2261 participants, the majority of whom were men, with a mean age of approximately 47 years. All participants were abstinent from alcohol prior to randomization, either as a result of completing a treatment program for alcohol dependence or by voluntarily engaging in an abstinence challenge—a structured campaign encouraging individuals to abstain from alcohol for 30 days, typically involving online registration and access to support and informational resources. Among the digital interventions used to support the abstinence maintenance, five were text-message based (Agyapong et al., 2012; O'Reilly et al., 2019; Haug et al., 2015; Agyapong et al., 2018; Lucht et al., 2021), three were app-based (Farren et al., 2022; Gustafson et al., 2014; Mellentin et al., 2019), one combined telephone and text-message support (Graser et al., 2021), one delivered an e-book self-help guide via email (Esselink et al., 2024), and one used a wireless breathalyser with facial recognition (Buono et al., 2023). Five different types of comparators were used, with treatment as usual being the most common (*n* = 8).

**Table 1:** – Baseline characteristics of the RCT included.

Author (year), country	Population (number (N), % sex, mean age)	Baseline treatment	Intervention, duration, and follow-up	Control
Agyapong et al (2012), Ireland (Agyapong et al., 2012)	*N* = 54 46.3 % Male 48.6 years	4-week inpatient dual diagnosis treatment programme	Twice daily supportive text messages for 3 months; 6 month follow up	Fortnightly text messages thanking for participating
Agyapong et al (2018), Canada (Agyapong et al., 2018)	*N* = 59 74.6 % Male 40.6 years	28-day residential addiction treatment programme	Twice daily supportive text messages for 3 months	Fortnightly text messages thanking them for participating
Buono et al (2023), USA (Buono et al., 2023)	*N* = 75 76 % Male 48.6 years	ARC In-home addiction treatment	Wireless breathalyser with facial recognition. 2 tests per day for 3 months with 6 week-follow up	TAU
Esselink et al (2024), The Netherlands (Esselink et al., 2024)	*N* = 602 44.3 % Male 57.12 years	TAC campaign	NoThanks self-help guide (e-mail) providing information, skills, and the motivation for behaviour changes; 5 chapters, 1 per week. 8 months follow-up	TAC as usual
Farren et al (2022), Ireland (Farren et al., 2022)	*N* = 111 52.3 % Male 45.8 years	30-day inpatient rehabilitation programme	Smartphone app UCD consisting of 12 CBT sessions, supportive text messages twice daily, Drinking and recovery logs, Personalised craving intervention, Gamification (reward-based behaviour modification technique); 6 months follow-up	TAU - weekly 90-minute therapist-led support groups, 60-minute lecture on addiction, and individual psychiatric interventions when necessary
Graser et al (2021), Switzerland (Graser et al., 2021)	*N* = 240 66.7 % Male 50 years	12-week abstinence-orientated residential treatment programme	Either high (10 contacts) or low (3 contacts) CBT TEL intervention or a 10-contact TEX (behavioural self-monitoring techniques); 6 month follow up	1 contact only and were not contacted until follow up
Gustafson (2014), USA (Gustafson et al., 2014)	*N* = 349 60.7 % Male 38.3 years	Residential Action treatment Programme	A-CHESS App and TAU for 8 months; 12 months follow up	TAU
Haug et al (2015), Switzerland (Haug et al., 2015)	*N* = 50 76 % Male 47.1 years	Outpatient alcoholic treatment centre	6-month after care programme with motivational text messages to stick to goals and proactive telephone calls from counsellors for support; 6 months follow-up	TAU
Lucht et al (2021), Germany (Lucht et al., 2021)	*N* = 462 77.3 % Male 45 years	Inpatient detoxification programme	SMS intervention (automated messages sent to patients over 1 year, which could trigger a phone call from a therapist) + TAU	TAU
Mellentin et al (2019), Denmark (Mellentin et al., 2019)	*N* = 164 77.4 % Male 46.3 years	Standard 3-month primary treatment at the outpatient clinic	CET as group aftercare or CET as fully automated mobile phone app for 8 weeks; 6 months follow-up	TAU
O'Reilly et al (2019), Ireland (O'Reilly et al., 2019)	*N* = 95 46.3 % Male 48.1 years	30-day rehabilitation programme	Twice daily supportive text messages over 6 months + TAU; 6-month follow-up	TAU

A-CHESS: Addiction-Comprehensive Health Enhancement Support System; ARC: Aware Recovery Care; CBT: cognitive behavioural therapy; CET: Cue exposure therapy; SMS: Short Message Service; TAC: temporary alcohol abstinence challenge; TAU: treatment as usual; TEL: telephone based; TEX: text message; UCD: “UControlDrink”.

The outcomes of all RCTs were related to maintain abstinence by measuring abstinence days or time to first drink. Among the five studies using text message interventions, only 2 (40 %) reported significant improvements in abstinence (O'Reilly et al., 2019; Lucht et al., 2021); however, these effects did not persist beyond six months (O'Reilly et al., 2019). Of the three studies evaluating smartphone app-based interventions, two (67 %) showed an increase in abstinence days (Farren et al., 2022; Gustafson et al., 2014). However, in the third trial (Mellentin et al., 2019), all participants showed a reduction in abstinence following discharge from outpatient treatment, suggesting that the intervention was ineffective. Nevertheless, there was no evidence of harm, as relapse—defined by the loss of abstinence—is a common indicator of treatment failure in AUD. The remaining trials, which assessed interventions using a wireless breathalyser (Buono et al., 2023), a self-help e-book delivered via email (Esselink et al., 2024), and a combination of telephone and text message support (Graser et al., 2021), did not demonstrate significant improvements in abstinence days or overall sobriety. A summary of the trials findings is presented in Table 2.

**Table 2:** Summary of findings from the RCTs.

Author (year)	Outcomes	Results	Summary
Text message based			
Agyapong et al (2012)	CAD	There was a trend for a higher CAD in the intervention group 88.3 (6.2) than control 79.3 (24.1), *t* = 1.78, df = 48, *p* = 0.08. Continuous abstinence from alcohol was observed in 83.3 % of the participants in experimental group compared to 61.5 % on control group.	Although not significant, the intervention showed a trend for a higher CAD than the control group
Agyapong et al (2018)	CAD	No significant difference in the mean CAD between the groups at 3 months (*p* = 0.25), but there was a trend for longer CAD in the intervention group 83.5 (19.3) than the control group 73.6 (33.0)	Although not significant, the intervention showed a trend for longer CAD than the control group
Haug et al (2015)	AUDIT-C	Rates of at-risk alcohol use (AUDIT-C ≥ 4) at the follow-up interview was 41.7 % (n = 10/24) in the control group and 28.6 % (n = 6/21) in the intervention group (OR = 0.56, 95 % CI = 0.16–1.95, *p* = 0.36).	The initial effectiveness test was not significant in maintain sobriety.
Lucht et al (2021)	Abstinence	There was more abstinence days reported during 1 year after randomization in experimental group than in the TAU group (on average, 267 vs 242; *p* = 0.037); Abstinence at 10–12 months follow up: Interventio*n* = 104 vs Control = 98. OR(95%CI): 1.68 (1.11–2.54) *p* = 0.035	Patients in the intervention group (SMS + TAU) reported a significantly higher number of abstinence days
O'Reilly et al (2019)	DDs	Between baseline and 3-month there was no significant difference between groups; At 6-months the intervention group showed a greater reduction in alcohol consumption in comparison to control participants from baseline (U = 494, *p* = 0.03, *r* = −0.3); At 6 months post-treatment follow up, there was no significant difference between groups in the number of drinking days (U = 551, *p* = 0.4, *r* = 0.1), or UPDD (U = 528, *p* = 0.47, r = 0.1);	The positive impact on alcohol consumption took longer to emerge, and these benefits did not persist after cessation of text messages

Smartphone App			
Farren et al (2022)	DDs	The mean (95%CI) number of DDs for the APP at baseline was 57.5 (50, 65.1; *p* < 0.05), at 3 months was 2.8 (0.34, 5.2; *p* < 0.05), and at 6 months was 1.8 (−0.26, 4; *p* > 0.05). For the TAU group, the number of DDs at baseline was 70.1 (62.8, 77.4; p < 0.05), at 3 months was 3.2 (1, 5.5; p < 0.05), and at 6 months was 2.7 (0.66, 4.7; p < 0.05).	Both groups had lower drinking days at the end of the 6 months, but the control group had more drinking days than the intervention group at each follow up
Gustafson et al (2014)	DDs	Intervention group had significantly less Risky drinking days than control group Overall (*p* = 0.003), at 4 months (*p* = 0.02), and at 12 months (*p* = 0.032), but not at 8 months (*p* = 0.096)	Intervention group had significantly lower number of risky drinking days
Mellentin et al (2019)	Abstinence	Significant decrease in abstinence between pre- to post-aftercare, PMC -0.12, SE 0.04, z = −3.15, *p* = 0.002; Significant decrease in abstinence between pre-aftercare to 6 months follow up, PMC -0.27, SE 0.06, z = −4.92, *p* < 0.001	Participants' alcohol consumption and rates of relapse increased following treatment

Wireless breathalyser			
Buono et al (2023)	AASE	The main effect for temptation (F (1,68) = 15.397, *p* < 0.01, η (Bickel et al., 2024) = 0.185), indicated that temptation significantly faded over time for both groups	Although not significant, the experimental group was higher than TAU in confidence score at a follow-up (13.78 vs 11.92; *p* = 0.142)

Email self-help guide (e-book)			
Esselink et al (2024)	Successful abstinence	Participants of the experimental group did not have a greater chance of completing the TAC successfully than participants of the control group (OR = 0.83, CI = 0.59–1.16; *p* < 0.05)	No statistical significance on maintain abstinence between self-help guide and TAU

Telephone and text message			
Graser et al (2021)	Abstinence and time to first drink	Abstinence at 6 months follow up: High TEL = 57 %, Low TEL = 48 %, TEX = 46 %, Control = 36 %. Control group had significantly more relapses than high frequency TEL (X2 = 5.02, df = 1, *p* < 0.05). There was no significant difference in abstinence between all other groups. 37.8 % relapsed within the first 4 weeks and 72.7 % within 3 months following discharge. There was a trend for shorter time to first drink in control group than high frequency TEL (log-rank test: X2 = 3.09, df = 1, *p* = 0.079) but this was not significant. No significant difference in all other groups time to first drink	High frequency TEL had the highest abstinence rates. There was no significant difference in time to first drink (relapse)

AASE: Alcohol Abstinence Self-Efficacy Scale; AUDIT-C = Consumption items of the Alcohol Use Disorders Identification Test; CAD: Cumulative abstinence duration in days; CI: confidence interval; DDs: drinking days; OR: Odds ratio; PMC: predicted mean change; SD: standard deviation; TAC: temporary alcohol abstinence challenge; TAU: treatment as usual; TEL: telephone based; TEX: text message.

### 3.3 Quality assessment

**Fig. 2:**
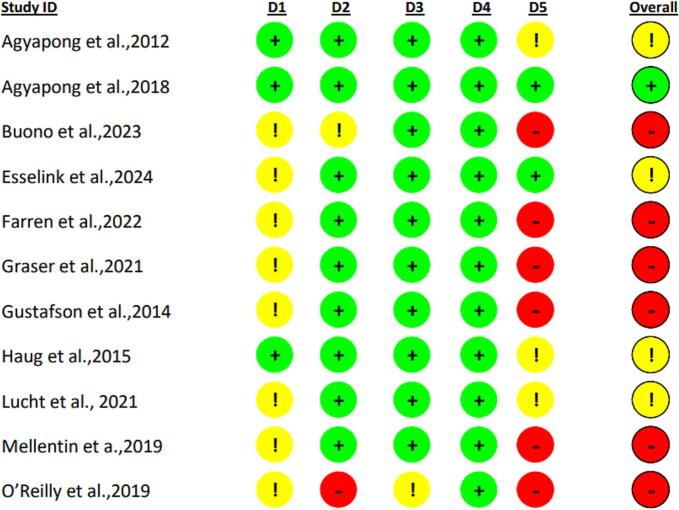
Risk of bias assessment using RoB 2.0. D1: Randomization process; D2: Deviations from the intended interventions; D3: Missing outcome data; D4: Measurement of the outcome; D5: Selection of the reported result.

Quality assessment analysis using RoB 2.0 identified a moderate to high risk of bias in all studies except one (Agyapong et al., 2018). The primary concerns were related to the randomization process, particularly the lack of information on allocation concealment before the start of the trial. Additional issues included the use of multiple scales, several time measurements, diverse outcome analyses, and reliance on self-reported measures. The results of the quality assessment are illustrated in Fig. 2.

## 4 Discussion

This systematic review examined the available evidence on digital interventions designed to support alcohol-dependent individuals in maintaining abstinence after discharge from an AUD treatment program. The included studies evaluated a range of digital interventions, including telephone-based support (calls and text messages), mobile apps, wireless breathalysers, and self-help e-books.

Among the 11 RCTs included, only four demonstrated statistically significant results (Farren et al., 2022; O'Reilly et al., 2019; Gustafson et al., 2014; Lucht et al., 2021). However, nearly all studies showed a quantitative positive effect compared to the control group. One RCT reported a negative association following the intervention for all participants, which may have been due to the low effectiveness of the primary AUD treatment. Overall, there is insufficient evidence to establish a strong link between digital interventions and sustained abstinence after AUD treatment discharge. However, further research in this area could provide valuable insights.

Enhancing aftercare for AUD through digital interventions has the potential to reduce the burden on healthcare systems and expand access to support, particularly by allowing individuals to engage privately through their own devices. Digital tools may also help reach individuals who might otherwise avoid treatment due to barriers such as social stigma. Evidence from this systematic review suggests that the effectiveness of digital interventions is greater when they incorporate multiple components—such as cognitive behavioural therapy (CBT)-based apps, craving management tools, and therapist contact—particularly when implemented following intensive inpatient treatment. In contrast, interventions relying solely on passive text messaging or general self-help content tend to show limited effectiveness, especially in studies with robust control conditions or broader, less clinically defined populations.

Therefore, the variation in significant and non-significant results across studies may be explained by differences in intervention complexity, participant engagement, and study design. Additionally, the effectiveness of the initial AUD treatment likely influenced post-discharge outcomes, with weaker primary care undermining aftercare benefits. Smartphone apps appear to have the greatest impact on supporting abstinence maintenance. A recent systematic review evaluating the effectiveness of smartphone interventions as continuing care for substance use disorders found that app-based interventions can serve as an effective alternative to traditional forms of aftercare, supporting the findings of our systematic review (Ramadas et al., 2023). Smartphone apps can integrate various strategies, such as remote cognitive behavioural therapy (CBT), educational and supportive content through reading materials, images, and videos, timely reminders, and peer interaction. This combination of digital tools may enhance the effectiveness of interventions compared to text-message-only approaches. Among the text-message-based interventions, Lucht et al. (2021) demonstrated a more prominent effect compared to O'Reilly et al. (2019). Lucht's study included therapist phone calls and referrals to additional services when needed, which extended the intervention beyond simple digital messaging. In contrast, O’Reilly's study was based solely on automated text messages.

Existing literature supports the use of digital CBT as an effective treatment for reducing alcohol and drug use overall (Gregory et al., 2024). Another systematic review found that, although the effect size was small and study quality was limited, app-based interventions may help enhance mental health treatments by reducing symptoms of depression, anxiety, mania, smoking, and alcohol use (Fuhrmann et al., 2024). Since these studies indicate improvements across different outcome measures, a combination of intervention types may be necessary to address the complex needs of individuals with AUD after treatment discharge.

This systematic review had limitations. By focusing solely on outcomes related to abstinence and sobriety, this review may have overlooked potentially valuable findings regarding the mental health benefits of digital interventions. This could have introduced bias toward abstinence-related outcomes reported in the trials, while ignoring other positive effects. Additionally, substantial differences in baseline characteristics across studies—including prior treatments, outcome definitions, follow-up durations, participants' mental health status, and co-occurring substance use—may have influenced the results. Furthermore, all participants in this review had completed an AUD treatment program before randomization, and the majority were male. Although gender and age were representative for the AUD general population (WHO, 2024), these findings may not be generalizable to non-treatment-seeking individuals or female populations with alcohol dependence. Another key consideration is the age of participants, as older individuals may face challenges in engaging with digital interventions. Moreover, the studies exhibited considerable heterogeneity in terms of comparators, outcomes, and sample sizes. Finally, the moderate to high risk of bias across the studies undermines the reliability of the findings.

Future research should aim to address the methodological limitations identified in this review by incorporating more rigorous study designs, including larger and more diverse populations, longer follow-up periods, and standardized outcome measures. As current evidence is insufficient to draw definitive conclusions, especially given the moderate to high risk of bias in existing trials, future studies should prioritize the use of objective clinical markers, such as biological tests for alcohol use alongside self-reported data to enhance validity. Additionally, there is a clear need to explore the effectiveness of digital interventions among underrepresented groups, including women, older adults, and individuals who have not completed formal treatment programs. Given the potential of app-based interventions to deliver integrated support combining cognitive behavioural therapy, educational content, reminders, and peer interaction, research should also investigate multi-component digital solutions. Combining the most effective features of different digital tools, such as smartphone apps and SMS-based interventions, may lead to more robust and scalable approaches tailored to the complex needs of people with AUD. Finally, the scope of future studies could be expanded to include mental health outcomes providing a more holistic understanding of the impact of digital aftercare interventions and support their use in broader addiction recovery strategies.

## 5 Conclusion

Despite its limitations, the evidence supporting digital interventions for continuing care in AUD rehabilitation remains valuable. Digital interventions can be effectively delivered through various modalities, including mobile applications, telephone-based programs, and online CBT. This enhances accessibility and allows users to choose the approach that best suits their needs. Additionally, these findings provide insights to guide future research. For instance, since both text message and app-based interventions have shown promise in maintaining abstinence, combining their most effective features may result in a more comprehensive intervention capable of addressing the complex needs of individuals with AUD more effectively than standalone approaches.

To further improve the quality of future studies and reduce the risk of bias, transitioning from subjective self-reported data to objective clinical measures—such as blood test readings—may be necessary.

## Funding

This research did not receive any specific grant from funding agencies in the public, commercial, or not-for-profit sectors.

## Declaration of competing interest

The authors declare that they have no known competing financial interests or personal relationships that could have appeared to influence the work reported in this paper.

## Appendix A Supplementary data

Supplementary material 1 Image 1

Supplementary material 2 Image 2
