# Supplementary material for: Digital interventions for supporting alcohol abstinence in aftercare – a systematic review
Source: Internet Interv. 2025 May 15;40:100832. doi: 10.1016/j.invent.2025.100832 (PMC12143608; doi:10.1016/j.invent.2025.100832)
Supplement: Supplementary file 2 — Supplementary material 2 [file mmc2.docx]

| **#** | **Query** |
| --- | --- |
| 1 | exp Alcohol-Related Disorders/ |
| 2 | "alcohol use disorder".tw. |
| 3 | (alcohol$ adj2 (drink$ or disorder or intoxicat$ or dependence or use$ or abus$ or addiction or addicted or misus$ or risk$ or consum$ or withdraw$ or detox$ or treat$ or therap$ or excess$ or reduc$ or cessation or intervention$ or rehab$ or depend$)).tw. |
| 4 | (drink$ adj2 (pattern$ or attitude$ or excess or heavy or heavily or harm or harmful or hazard$ or binge or problem$)).tw. |
| 5 | (alcoholic or alcoholism).tw. |
| 6 | exp Alcohol Drinking/ |
| 7 | *internet/ or *Computers/ or *Microcomputers/ or *digital technology/ or *online game/ or *social media/ or *Cellular Phone/ or *smartphone/ or *digital technology/ or *telehealth/ or *telemedicine/ or *digital intervention$/ or *virtual reality/ |
| 8 | *Therapy, Computer-Assisted/ or *computer assisted therapy/ or *Computer-Assisted Instruction/ or *computer mediated communication/ |
| 9 | *Blogging/ or *Social Media/ or *mobile application/ or *mobile phone/ or *mobile devices/ or *Electronic Mail/ or *websites/ |
| 10 | (telephone intervention or web-based).tw. |
| 11 | (Digital Health Resources or Digital Mental Health Resources).ti,ab,kw. |
| 12 | ((email$ or e-mail$ or electronic mail$ or text messag$ or SMS or MMS or phone? or cellphone? or cell-phone? or smartphone? or smart-phone? or digital tablet? or pda or personal digital assistant? or social media or social networking or facebook or twitter or skyp$ or zoom or app?) adj3 (deliver$ or generat$ or based or provid$ or facilitat$ or support$ or treatment? or therap$ or intervention? or program$ or feedback)).ti,ab. |
| 13 | ((Internet$ or electronic$ or digital$ or technolog$ or online or on-line or computer$ or laptop? or software or web$ or weblog$ or blog$) adj3 (deliver$ or generat$ or based or provid$ or facilitat$ or support$ or treatment? or therap$ or intervention? or program$ or feedback)).ti,ab. |
| 14 | (e-BI or e-SBI or ehealth or e-health or electronic health or mhealth or m-health or mobile health or virtual health or digital health or technological aid?).ti,ab. |
| 15 | (randomized controlled trial or controlled clinical trial).pt. or randomized.ab. or randomised.ab. or randomly.ab. or trial.ab. or groups.ab. |
| 16 | 1 or 2 or 3 or 4 or 5 or 6 |
| 17 | 7 or 8 or 9 or 10 or 11 or 12 or 13 or 14 |
| 18 | 15 and 16 and 17 |
